# Transformation of Context-dependent Sensory Dynamics into Motor Behavior

**DOI:** 10.1371/journal.pcbi.1002908

**Published:** 2013-02-14

**Authors:** Roberto Latorre, Rafael Levi, Pablo Varona

**Affiliations:** 1 Grupo de Neurocomputación Biológica, Dpto. de Ingeniería Informática, Escuela Politécnica Superior, Universidad Autónoma de Madrid, Madrid, Spain; 2 The Whitney Laboratory for Marine Bioscience, University of Florida, St. Augustine, Florida, United States of America; University College London, United Kingdom

## Abstract

The intrinsic dynamics of sensory networks play an important role in the sensory-motor transformation. In this paper we use conductance based models and electrophysiological recordings to address the study of the dual role of a sensory network to organize two behavioral context-dependent motor programs in the mollusk *Clione limacina*. We show that: (i) a winner take-all dynamics in the gravimetric sensory network model drives the typical repetitive rhythm in the wing central pattern generator (CPG) during routine swimming; (ii) the winnerless competition dynamics of the same sensory network organizes the irregular pattern observed in the wing CPG during hunting behavior. Our model also shows that although the timing of the activity is irregular, the sequence of the switching among the sensory cells is preserved whenever the same set of neurons are activated in a given time window. These activation phase locks in the sensory signals are transformed into specific events in the motor activity. The activation phase locks can play an important role in motor coordination driven by the intrinsic dynamics of a multifunctional sensory organ.

## Author Summary

How sensory information is transformed into effective motor action is one of the most fundamental questions in neuroscience. As this question is difficult to assess experimentally, biophysical models of sensory, central and motor systems contribute to understand the information processing mechanisms involved in this transformation. Biophysical models can be informed by electrophysiological data in those situations where it is possible to record neural activity at all stages of sensory-motor processing. In this paper we use this approach to describe the dual dynamics of a multifunctional sensory organ in the mollusk *Clione limacina* and its transformation into two different motor programs. Our experimental and modeling results indicate that the sensory signals are modified to fit the changing behavioral context, and they are readily interpreted by the rest of the nervous system to produce the correct motor output.

## Introduction

One of the most fundamental questions in neuroscience is how sensory information is transformed into effective motor action. This question is difficult to assess experimentally as it implies monitoring neural activity at different stages of the sensory-motor transformation, a task that can be more easily addressed in simple animals [1]–[4]. Experimental evidence points to the important role that intrinsic sensory dynamics, i.e., neural dynamics that does not directly correlate to the dynamics of a physical external stimulus, can serve in this transformation. In particular, there are several examples from neurophysiological studies that show complex intrinsic dynamics in sensory networks [5], [6]. In most cases, the dynamics observed is directly related to the information encoding mechanisms in these systems.

Complex intrinsic dynamics can also be related to multifunctionality, which has only been partially addressed in neuroscience research, mainly in motor networks [7]–[13], with a few examples in sensory systems [14]. One remarkable example of relationship between intrinsic sensory dynamics and multifunctionality has been discussed for the gravimetric organ of the mollusk *Clione limacina* [15], [16].

**Figure 1:**
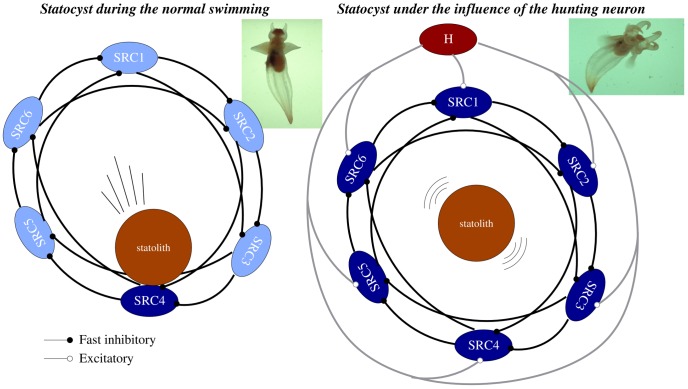
The statocyst model. A network of six statocyst receptors (SRC1-6) used in our experiments to model a single statocyst. Each SRC is connected to the next two adjacent cells. The sensory network controlling routine swimming (left panel) and hunting (right panel) is the same. During routine swimming, the only source of excitation for the SRCs is the statolith, whereas during hunting the interneuron H adds significant excitation to all sensory neurons. Dark blue indicates the SRCs receiving excitatory inputs in each case.

The marine mollusk *Clione limacina* (see insets in Figure 1) is a predator whose only prey is another mollusk, *Limacina helicina*. Because of the simplicity of its nervous system, this animal is a well known model to study both sensory and motor processing, and thus the transformation that occurs between them. During routine swimming, when water disturbance changes its head-up body orientation (see the left inset of Figure 1), *Clione* tries to correct for the change by actively moving the wings and the tail [17]. Several neural structures are involved in the control of the orientation of *Clione*'s body during this routine swimming [17]–[19]. As the main sensory input, *Clione* uses its gravimetric organs, the statocysts. These are a pair of spherical structures located in the pedal ganglia which contain a stone-like structure –the statolith– that moves under the effect of gravity. The statolith exerts pressure on the internal wall of the sphere which is lined with statocyst mechano-receptor cells. The statocyst receptor cells (SRCs) react to the pressure of the statolith allowing the animal to determine changes in the orientation of its body. Sensory information about the orientation of the body is sent from the mechano-receptors to several groups of cerebro-pedal interneurons. These interneurons in turn control the central pattern generators that drive *Clione*'s wing and tail motoneurons, which add steering (i.e., to induce transient corrective motions in the wings and tail to achieve the preferred head-up position during routine swimming) [18], [20]–[23]. In addition to gravimetric signals, chemical sensory information about the presence of prey is conveyed to the SRCs through excitatory input from a pair of cerebral hunting interneurons (CHI) [24], [25]. *Clione* does not have a visual system and although its chemosensors can detect the presence of *Limacina*, they are presumably nondirection-sensitive. When hunting behavior is triggered, the resulting hunting search consists of loops and turns in a complex trajectory to locate the prey. A quantitative analysis of the hunting search trajectories has been described in [15]. Hunting behavior typically occurs in different search episodes with resting times in between. The Videos S1 and S2 in the supplementary material illustrate *Clione's* typical routine swimming and hunting search behaviors. The two behavioral contexts described above make *Clione* a good animal model to study the mechanisms involved in sensory-driven motor activity.

There is a long tradition in building models of sensory-motor transformation based on electrophysiological recordings (e.g. see [26], [27]). In most cases, the sensory network is not included in the model as the intrinsic sensory dynamics are not taken into account. However, more complete computational models of the sensory-motor transformation can largely contribute to the understanding of how a given neural motor pattern is generated from sensory activity. In particular, theoretical efforts help to characterize the dynamics of sensory networks and to identify relevant features that are used in the organization of motor activity. We have previously developed a rate model to describe how a winnerless competition dynamics can arise within an inhibitory network [28] and pointed out its possible role in the organization of the complex hunting behavior of *Clione* [16], [29], [30]. Several predictions of the model regarding this type of intrinsic sensory dynamics have been tested in neurophysiological experiments: (i) the statocyst network produces a complex sustained spatiotemporal dynamics during hunting search behavior even when there is no motion in the *in vitro* experimental conditions, in contrast to the situation during routine swimming in which only a few neurons are active; (ii) during hunting behavior, the activity of the sensory neurons is correlated to the wing and tail motoneurons [15], [16].

In this paper we build a conductance based-model of the statocyst sensory network and the wing CPG. Our model illustrates how the multifunctional nature of the sensory network can drive the CPG activity in two different behavioral contexts. On one hand, the winner take-all dynamics in the gravimetric sensory network model is read by the wing CPG network and produces the steady rhythm during routine swimming. On the other hand, the same sensory network under a different stimulation produces a winnerless competition dynamics which is read by the motor network and results in the irregular patterns characteristic of wing motoneurons during hunting behavior. The model demonstrates that the spatiotemporal pattern of the sensory dynamics may be in the form of specific activation phase locks that emerge during hunting. These phase locks are transformed into specific motor events in the wing CPG model.

## Results

### Dual Sensory Dynamics

**Figure 2:**
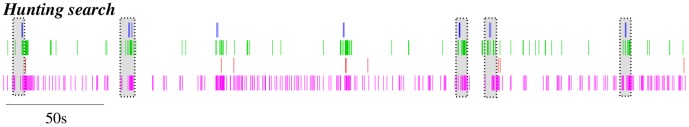
A representative recording of SRC firing activity in the biological system. Spikes times of four SRC units during fictive hunting search behavior evoked by physostigmine *in vitro*. The activity was recorded extracellularly and the spikes were sorted as explained in [16]. Note the irregular sequential alternation in firing between different SRCs during hunting. During these experiments there is no motion and thus the WLC activity arises from the excitation of the hunting neuron to the SRCs. Grayed areas illustrate specific sequential activations among active SRCs.

Experimental recordings have shown that during routine swimming, *Clione* uses information from the statocysts regarding body orientation to keep its preferred head-up posture (see inset in Figure 1, left panel) [21]. During this behavioral state a winner take-all (WTA) competition occurs between the SRCs and only the cell or group of cells pressed by the statolith persistently fire (see Figures 4 and 5 in [19]). This is in part due to the inhibitory nature of this network. The activated SRCs inform the rest of the nervous system about *Clione*'s body orientation and, if a postural change occurs, wing and tail activity generates transient corrective motions to recover the preferred head-up position. On the other hand, during the hunting search behavior a sustained winnerless competition emerges in the statocyst network (see Figure 2) which consists of an irregular alternation of firing among the SRCs. The presence of *Clione*'s prey evokes excitation of the CHI neurons, the *hunting neurons*, which send an excitatory input to the SRCs. When CHI neurons are activated, hunting behavior starts [24], [31], [32]. Hunting behavior can be evoked *in vitro* by applying the acetylcholinesterase inhibitor physostigmine to the animal (see “Methods and Models” section). In these experiments there is no motion and the main input to the SRCs comes from the external excitation of the hunting neuron and not exclusively from the statolith.

**Figure 3:**
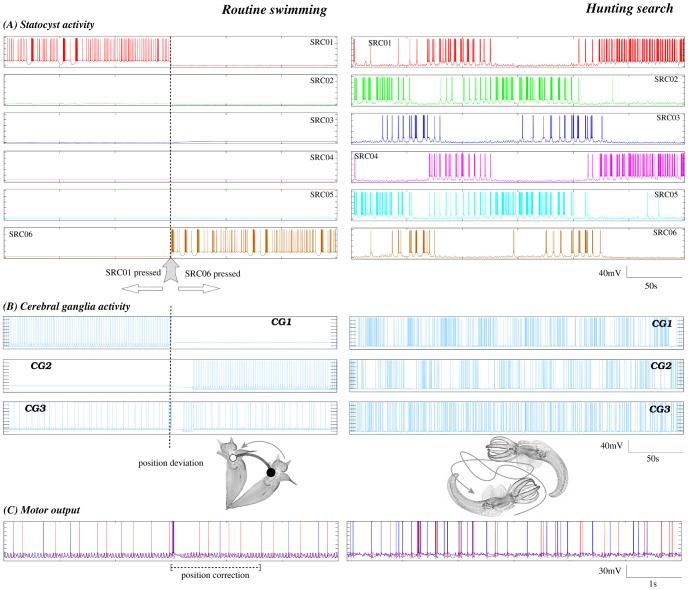
The behavior of the model network. Left panels: Routine swimming activity pattern, in the absence of the hunting neuron excitation (c.f. Figure 1, left panel). Right panels: Search hunting behavior activity pattern when the hunting neuron is activated (c.f. Figure 1, right panel). (A) Behavior of the SRC model network. During routine swimming the model statocyst network shows a winner take-all dynamics. Only the SRC pressed by the statolith fires (and thus inhibits other SRCs connected to it). In the first part of the time series, the active neuron in the statocyst network is SRC01. A simulation of body orientation change by exciting SRC06 (denoted by the arrow) makes the new pressed SRC fire immediately while SRC01 becomes silent like the rest of the SRCs. The winnerless competition dynamics (WLC) appears in the statocyst model when the hunting neuron is activated. The irregular sequential activations of the WLC activity displayed by the model are very similar to the one observed in the biological circuit (see Figure 2). (B) Behavior of the CG network located between the statocyst and the wing CPG. During routine swimming either CG1 or CG2 is active, depending on which of the SRCs receives the excitation from the statolith. The CG3 cell integrates and sends this information to the CPG. When a deviation from the preferred position occurs, the identity of the active SRC pressed by the statolith changes and the cerebral interneurons interpret this change (see panel C). During hunting, both CG1 and CG2 cells are active. (C) Behavior of the wing CPG model responsible for generating the periodic wing beating rhythm. As in the biological circuit (Figure 6) the activity of the wing CPG model is affected by the statocyst dynamics which is evoked by the behavioral context. During routine swimming, the model CPG generates a regular rhythm. During the hunting behavior the pattern is faster and highly irregular. Note that during routine swimming, when a change of posture is simulated, the sensory information received in the wing CPG immediately generates a corrective activity. Here, for example, the change from SCR01 to SCR06 produces a short change in the frequency and shape of the rhythm. Afterward, the typical repetitive rhythm is restored.

To model the gravimetric sensory network dynamics in these two different behavioral contexts (routine swimming and hunting behavior), we have used an inhibitory neural network with six SRCs under the action of the statolith and a CHI neuron (see Figure 1). Each cell of the network is implemented with a Komendantov-Kononenko conductance based model [33], a well characterized model for molluscan neurons that can qualitatively reproduce the spiking and spiking bursting behavior observed in *Clione*'s neurons. The sensory network is built with an asymmetric inhibitory connection topology inspired by the current knowledge of the statocyst network [15], [28]. A detailed description of this topology and the parameters used in our simulations, for both the individual behavior of each cell and the connectivity, can be found in the “Methods and Models” section. During the simulation of routine swimming with our statocyst model, the CHI is silent and does not excite the SRCs. Thus, the only input that the statocyst neurons receive in this case is the excitation from the pressure of the statolith (simulated as a current injected in a specific SRC). The left panel in Figure 3A shows that in this situation, a winner take-all dynamics appears in the statocyst conductance-based network model, and only the SRC pressed by the statolith fires. A simulation of body orientation change as illustrated here by exciting another SRC (see arrow on the left panel of Figure 3A) causes a change of the active neuron in the statocyst network: the new pressed SRC starts firing immediately while the other neurons are silent.

**Figure 4:**
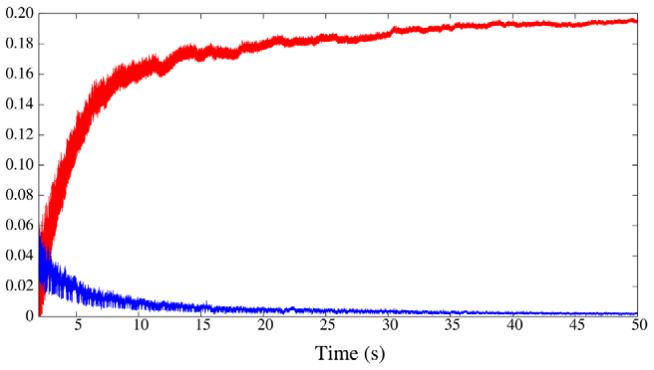
Lyapunov exponents calculated from WLC behavior. Evolution in the calculation of the two positive Lyapunov exponents in the statocyst model under the action of the hunting neuron. The existence of two positive Lyapunov exponents means that the activity in the network is chaotic during hunting behavior.

In our model network we simulate the presence of a prey by activating the CHI neuron, which excites all SRCs. This activates the hunting behavioral state within the same sensory network model (Figure 1, right panel). Under the CHI neuron excitation, the dynamics of the model sensory network changes to a winnerless competition (WLC) among the SRCs and the action of the statolith hardly affects the network dynamics (see Figure 3A, right panel). This competition arises from the hunting neuron excitatory input to all SRCs and the inhibitory connections within the network. In this WLC dynamics the SRCs display switching activations of varying durations including some overlappings, as the inhibition among the SRCs is moderate. The activity of the network in this case is highly irregular. These irregular sequential activations in the model are qualitatively similar to those observed in the biological network during fictive hunting behavior evoked *in vitro* (c.f. right panel of Figure 3A and Figure 2, where four SRCs from the same statocyst are recorded simultaneously in an *in vitro* preparation). The level of irregularity of the sensory network model can be characterized by calculating the Lyapunov exponents. Figure 4 shows the evolution of the calculation of the positive Lyapunov exponents from the vector field (see “Methods and Models” section for details) describing the sensory network during hunting behavior. The presence of two positive Lyapunov exponents (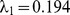 and 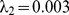) in this mode of operation indicates that this network activity is chaotic, which reflects the richness of its dynamics. No positive Lyapunov exponents exist in the analysis of the statocyst activity during routine swimming.

### Motor Activity

*Clione*'s sensory network has been studied in detail from a theoretical point of view using rate models, in particular the conditions to generate WLC dynamics [28], [30], [34]. Less attention has been paid to modeling the CPG responsible of the animal's movements and the transformation of the sensory dynamics into a motor activity. This can be addressed with more detailed biophysical models of neurons and synapses to better reproduce the connectivity and rhythms observed in the *in vitro* experiments.

**Figure 5:**
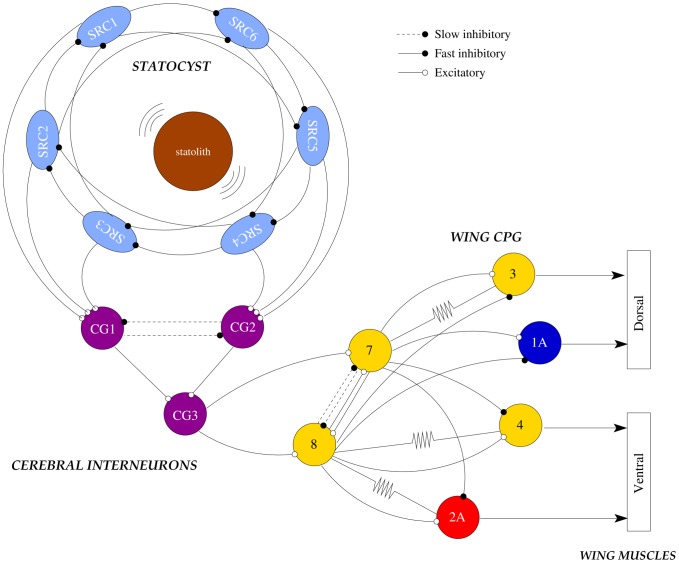
Statocyst model connected to a wing CPG model. Statocyst dynamics drive the motoneurons that control the movement of the wings. Here we have modeled the wing CPG circuit by building a network with six neurons: 7, 8, 1A, 2A, 3 and 4. This network replicates the known wing CPG connectivity 20,42. Each single neuron represents the equivalent electrically coupled groups of cells in the biological circuit. We have chosen neurons 1A (for the dorsal group) and 2A (for the ventral) as representative cells of the CPG behavior. The statocyst is connected to this CPG through a simple model of cerebral cells that consists of three cerebro-pedal interneurons (CG1-3). Note that we omitted the hunting neuron in the statocyst circuit diagram to simplify the graphical representation.

Experimental evidence shows a significant correlation between the activity of the SRCs and the wing CPG cells (e.g. see Figure 5 in [15]). The dynamics of these sensory and motor networks have very similar time scales. Thus, we have developed a simple cerebral interface (CG1, CG2 and CG3) between the statocyst and a model wing CPG (see Figure 5 and “Methods and Models” section for details). The model cerebro-pedal interneurons integrate the activity of the sensory network and relays it to the wing CPG. In this interface CG1 and CG2 inhibit each other to avoid contradictory simultaneous left and right signals from the SRCs. Figure 3B shows the behavior of the model cerebral interface in response to a winner take-all mode (left panel) and a winnerless competition behavior (right panel) in the statocyst conductance based model. During simulation of routine swimming, only the SRC pressed by the statolith is active. This results in the activation of CG1 or CG2. During the simulation of a deviation from the preferred head up position, illustrated in the left panel of Figure 3A, CG1 stops firing while CG2 becomes active (left panel of Figure 3B). The activity of CG3 is modulated by this change, which leads to a short temporal variation in the regular beating pattern generated by the CPG (see below). During hunting behavior, the statocyst generates a stronger activity in all SRCs and, consequently, increases the cerebral interneuron activity, which maintains the competition of the CG1 and CG2 cells with an irregular bursting pattern.

**Figure 6:**
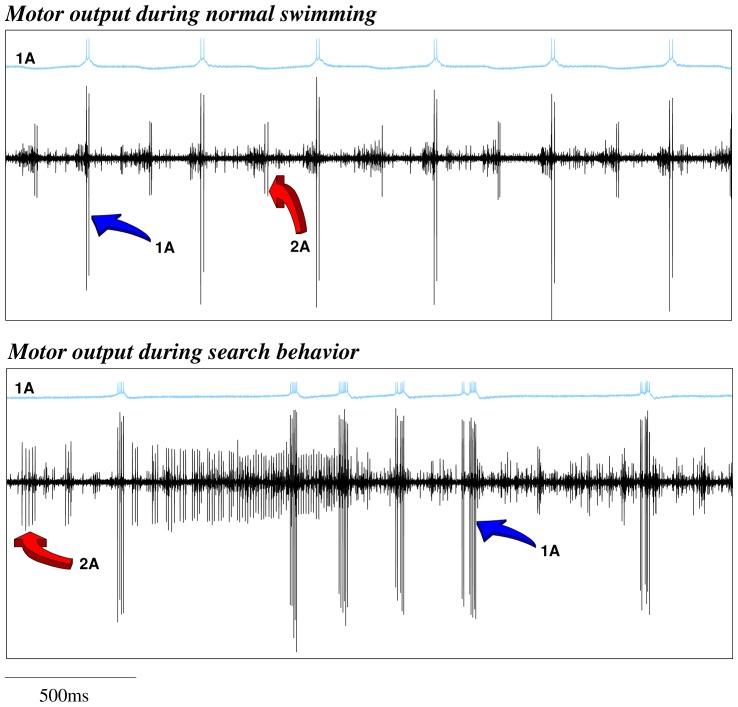
The behavior of the biological wing CPG. The wing CPG generates the rhythm that controls the wing movements. Each panel of the figure displays intracellular recordings of the 1A neuron (blue traces) and the extracellular activity of the wing nerve (black traces) during the two behavioral contexts: routing swimming (top panel) and search hunting (bottom panel). The arrows in the extracellular traces point out the activity associated with the firing of the 1A and the 2A cells. 1A and 2A neurons have been reported to fire doublets as well as single spikes depending on the strength of the swimming.

*Clione*'s wing CPG rhythm consists of the fire alternation of two half centers: the dorsal and ventral neural groups [18], [20]. In our CPG model (Figure 5) these groups are composed of three neurons. Each of these neurons represents an electrically coupled group of cells that fire synchronously. We use the same nomenclature as in [18]. The rhythm generators are neurons 7 and 8, while neurons 1A, 3, 2A and 4 are the motoneurons that innervate the wing muscles (see the network details in the “Methods and Models” section). All the neurons in the same group (7, 1A and 3 in the dorsal; and 8, 2A and 4 in the ventral) fire synchronously, so here we will analyze the rhythm generated by 1A and 2A cells. In the winner take-all mode of the model statocyst, the response pattern generated by the wing CPG model consists of the alternation of firing in interneurons 7 and 8 that, respectively, drive motoneurons 1A and 3 in the dorsal group; and 2A and 4 in the ventral group. This pattern in the models is similar to the one observed in *in vitro* electrophysiological recordings of *Clione*'s motoneurons during routine swimming (c.f. left panel in Figure 3C and top panel in Figure 6). Only small changes in the mean rhythm frequency can be observed depending on the SRC pressed by the statolith (Table 1). In this behavioral mode, motoneuron 1A is active in the dorsal phase, and 2A motoneuron is active in the ventral phase, thus driving the wing flapping [18], [20]. The left panel in Figure 3C illustrates the response of the CPG model to the simulation of a transient change in the *Clione*'s preferred orientation. In this situation, a fast change in the beating rhythm occurs. This fast motor response is always coherent with the change in the sensory network, i.e., the same sensory input (a specific change in the SRC pressed by the statolith) produces the same output in the CPG. These changes can presumably be translated into the wing movements required to generate the compensatory gravitational reflexes needed to correct small deviations in the animal's orientation [19], [21]. Note that, as can be observed in Video S1, the corrective beatings consist of successive dorsal or ventral movements. Immediately after that, the regular beating is restored.

**Table 1:** Mean frequency of the CPG rhythm during routine swimming.

SRC pressed	Dorsal frequency (Hz)	Ventral frequency (Hz)
SRC01	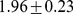	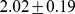
SRC02	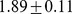	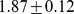
SRC03	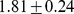	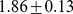
SRC04	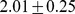	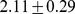
SRC05	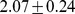	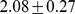
SRC06	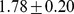	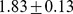

Frequency of the model wing CPG rhythm during routine swimming depending on the SRC pressed by the statolith. The rhythm generated is always the same and consists of the alternation of the dorsal and the ventral phases.

On the other hand, although the sequential activations in the statocyst model network are highly irregular during hunting, the wing CPG produces a coordinated rhythmic pattern that could generate the complex motion observed during hunting in the behavioral experiments. As in the biological network (c.f. right panel in Figure 3C and bottom panel in Figure 6), this rhythm is non-regular and at higher frequency than during the routine swimming [18], [20]. The calculation of the Lyapunov exponents from the vector field of the wing CPG model in this situation yields two positive Lyapunov exponents: 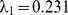 and 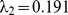. There are no positive Lyapunov exponents during the simulation of routine swimming. This means that the activity in the CPG model changes from regular to chaotic depending on the sensory context.

As Video S2 shows, hunting behavior in the living animals consists of different hunting episodes with resting times in between. Even though *Clione* generates a highly irregular sensory activity during these episodes, this activity has to be coordinated to be effective in driving the hunting search. During *in vitro* fictive hunting, specific activation sequences appear among the recorded SRCs (highlighted as grayed regions in Figure 2). The activity recorded during different hunting episodes in the physiological experiments can be classified into different types using a principal component analysis (PCA). In this representation, previous experimental results have shown that similar patterns in the sensory network induce similar patterns in the motor system [15], pointing to the significant correlation between the activity of the SRCs and the activity of the motor cells [15], [16]. To validate that our model reproduces these experimental results and assess how the different sensory activations are translated into motor commands, we used for the simulated data a PCA analysis similar to the one employed before for experimental recordings. With this kind of analysis we could display a high-dimensional dynamics in a three dimensional representation.

**Figure 7:**
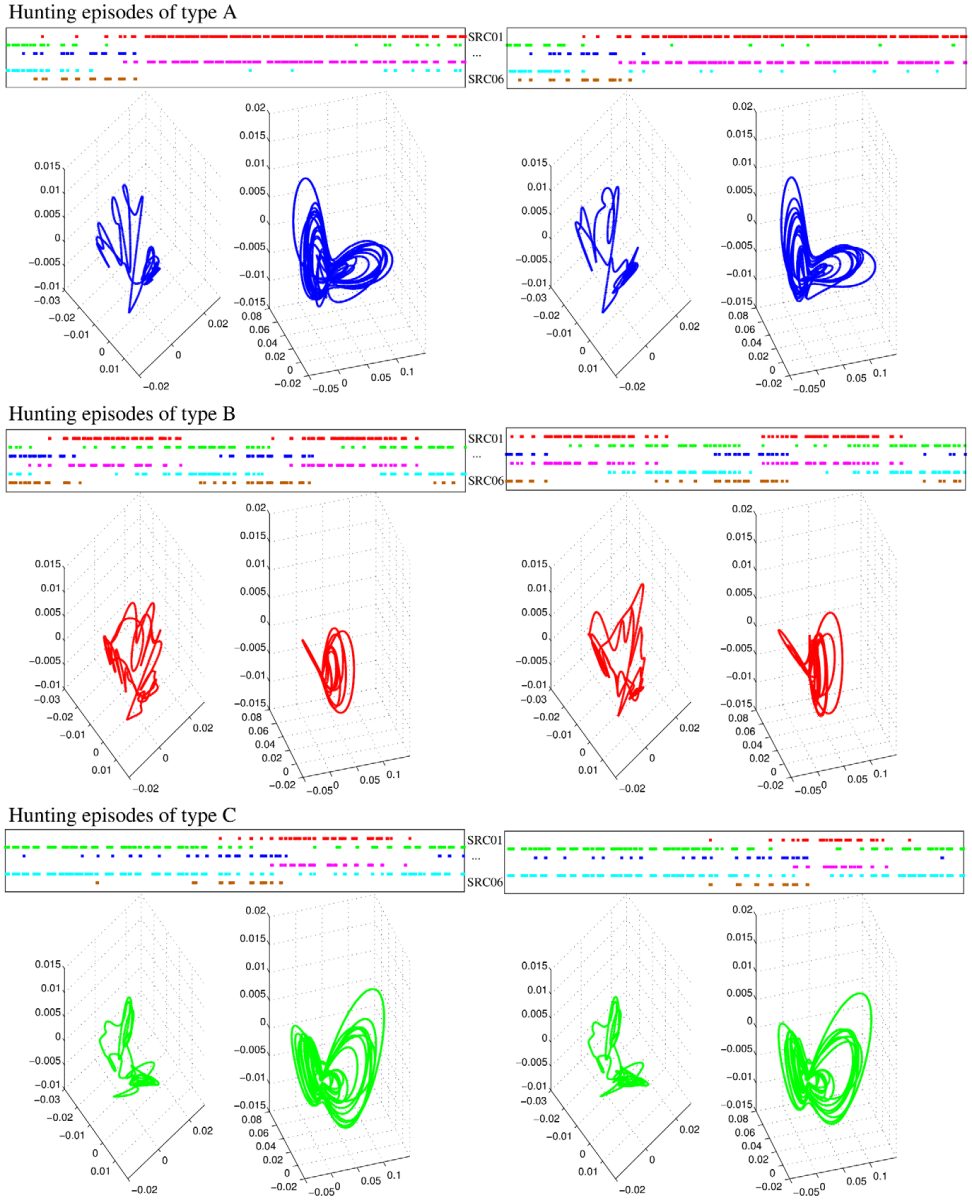
Principal component analysis of the sensory-motor transformation. We have selected three different types (A–C) of hunting episodes according to the duration of specific patterns of sequential activations among the SRCs. A total of six hunting episodes are shown in this figure, two examples for each kind. The top panels display the time intervals in which each neuron exceeds a threshold of 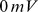. Different colors are used to indicate each neuron. The episodes labeled as type A correspond to long activations of neurons 1 and 4 (red and magenta, respectively). The episodes labeled as type B correspond to sequential activations of similar length in all neurons, while type C episodes correspond to long activations of SRCs 2, 3 and 5 (green, blue and cyan). The bottom panels display the first three principal components for the activity of the six receptor cells (left plot) and the four motoneurons in the wing CPG (right plot). The PCA shows that a similar sequential activity in the sensory network during hunting (different hunting episodes of the same type) evokes similar rhythmic activity in the motor system.

For our model analysis, we defined hunting episodes as time windows where similar sequences, in terms of the duration of specific patterns of sequential activations of the six SRCs, appear consecutively in the sensory dynamics at least three times. As an example, the top panels in each row of Figure 7 show three different representative types of hunting episodes in the statocyst model network. Type A corresponds to sequences of long activations of SRCs 1 and 4, and short activations of SRCs 2, 3, 5 and 6. Type B corresponds to sequential activations of similar length in all the SRCs. And type C corresponds to long activations of SRCs 2, 3 and 5, and short activations of SRCs 1, 4 and 6. In general, the specific activation pattern in the statocyst model during hunting arises from different departing activations of the SRCs and/or different CHI neuron inputs. However, if the simulation of hunting is long enough, the WLC dynamics in the statocyst network evolves to different activation patterns at different intervals. Therefore, in this situation, a given time series can contain hunting episodes of different types. For convenience, in our analysis of hunting behavior we used this approach while studying long time series of hunting. In these time series, first we identified and classified the different hunting episodes into different types according to the duration of specific patterns of sequential activations among the SRCs. Once different hunting episodes were identified, we analyzed the activity pattern of the model during these time windows with the PCA.

The first three principal components of the sensory and motor signals analyzed here explain more than 90% of the total variability of the signals, thus the PCA of these time series can be used to characterize the dynamics of the sensory and the motor network. The percentage of the variability explained by each of the three principal components is the following: first PC 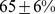, second PC 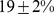; third PC, 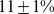 for the statocyst; and first PC 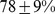, second PC 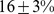; third PC, 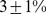 for the wing CPG. Bottom panels in each row of Figure 7 show the PCA representation of the activity for three examples of hunting episodes in the statocyst model network, as well as, the corresponding representation for the motor activity. Note that similar patterns in the statocyst model network evoke similar patterns in the motor network as observed in the experimental results [15].

**Figure 8:**
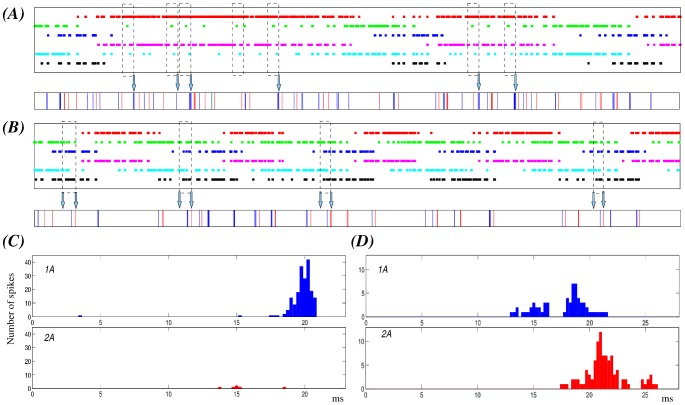
Specific sensory network activation phase locks correspond to a unique motor activity during hunting behavior. The figure illustrates two representative examples of activation phase-locks among activated SRCs in a given time window (dashed rectangles) and its corresponding motor output (arrows) during different hunting episodes. (A–B) Top panels: Statocyst sequential activation patterns. The color codes for the neurons are the same as in Figure 7. Bottom panels: Motor response to the sensory activity. Blue and red traces correspond, respectively, to the firing of 1A and 2A motoneuron. The analysis reported in the text refers to 120 s simulations but here, for representation purposes, we show a fragment of 12 s. The dashed rectangles point out the specific activation phase locks as statistically selected (see Methods) for time windows in which four specific neurons are active. Panel A displays activation phase locks for SRCs 1, 2, 4 and 5 during a hunting episode characterized by long activations for neurons 1 and 3. These specific activation phase locks result in similar responses in the motor network –see also panel C– in most cases (6 out of 7 in the example shown here). In the hunting episode of panel B the activations have a similar duration in all neurons. The sequences pointed out here involve SRCs 2, 3, 5 and 6. In this case, the sensory activation phase lock always induces the same response in the wing CPG –see also panel D–. (C) PSTHs of the 1A and 2A cells characterizing the motor response during the activation phase lock illustrated in panel A. (D) PSTHs of the same cells during the phase lock illustrated in panel B. PSTHs are calculated for the entire simulations (120 s). Spikes are aligned to the beginning of the activation sequence. The activation phase lock of panel A induces in most cases the firing of motoneuron 1A, which is not accompanied by activity in 2A. On the other hand, phase locks in panel B induce the firing of 1A followed by the activation of 2A in a similar interval.

In our simulations of hunting behavior, we observe that the irregularity of each hunting episode of the sensory network is built out of sequential switchings among the SRCs, with activation phase locks of different durations involving a set of neurons activated in a given time window. By identifying specific activation phase locks in the sensory network (Figure 8, dashed rectangles), we saw that they are transformed into a specific fast irregular beating command of the wing CPG. To assess the response of the wing CPG to each of the activation phase locks, we first detected them (with the method described in the “Methods and Models” section) and then we analyzed the corresponding motor output. The motor response was characterized by the peristimulus time histograms (PSTH) of 1A and 2A neurons. In this analysis we searched for activation sequences of at least four SRCs which appeared a minimum of 30 times in different time series of 120 seconds, and we aligned the motoneuron spikes to the beginning of the activation sequence to calculate the PSTHs (Figure 8). Although in this search for activation phase-locks we allowed the duration of the activity to be different, the sequence of the switching among the SRCs activated in a given time window was preserved. During hunting, the activity is chaotic both in the sensory and the motor model networks (characterized by two positive Lyapunov exponents). Nevertheless, in 88% to 100% of the cases depending on the specific activation phase lock, a given sequence in the SRC network produces the same stereotyped motor activity in the motoneurons of the wing CPG. Note that the sequential activations are not exactly the same in terms of duration and preceding activity, which may be the source of the small number of missing events. In panels A and B of Figure 8 we illustrate two representative examples of sensory activation phase locks and the corresponding motor response in different hunting episodes. In the first example (panel A), we show (dashed rectangles) the activation phase locks among SRCs 1, 2, 4 and 5 during a hunting episode where the activations for neurons 1 and 4 are long. The corresponding PSTHs in panel C of Figure 8 show that these specific sequential activations induce a strong activity in motoneuron 1A at the end of the activation sequence. This response is produced in 71 out of 74 sequences during a 120 s simulation (6 out of 7 in the time series shown in the figure). The second example of activation phase lock shows another episode in which the activations have a similar duration in all neurons. The sequences shown in this case involve SRCs 2, 3, 5 and 6. As the corresponding PSTHs in panel D of Figure 8 indicate, these sequential activations induce the firing of 1A followed shortly after by 2A (32 out 32 sequences during a 120 s simulation). The fact that specific sequential activations in the sensory network may be interpreted by the CPG and lead to specific events in the motor activity is something that can be used by the system for the coordination of the wing beating.

The dual dynamics of the statocysts in two different behavioral contexts (routine swimming and hunting behavior) are directly translated into the corresponding characteristic motor behavior. The same CPG that controls the periodic wing beating during routine swimming reacts to the irregular commands from the sensory network to generate the hunting motor program. During the winner take-all phases, the sensory gravitational input from the statolith is used by the motor system to react to small deviations from the preferred head up orientation by generating compensatory gravitational reflexes [19], [21]. This behavior is reproduced by our model. During routine swimming the wing CPG model generates a regular pattern of activity able to control the wing beating until a deviation is simulated. This generates a fast transient response that could produce the required correction movements. After that, the regular pattern starts again. On the other hand, the activity generated during the winnerless competition phases acts on the same network to shape the irregular but coordinated search motor behavior [15]. In this case, the CPG model produces a motor output activity that is able to drive this complex motion.

## Discussion

It is difficult to experimentally assess the study of all the stages present in a sensory-motor transformation. Because of the lack of experimental results, there are also very few models that address the transformation of sensory dynamics into a motor program. *Clione limacina* is an experimental model in which this study is possible. In this paper we have discussed the dual role of a sensory organ in relationship to two types of motor behaviors during routine swimming and hunting behavior. To address this issue, we have built neural network models ranging from a single statocyst network to a system where the activity of the statocyst is transferred to a motor wing CPG through a simple model of cerebral ganglia. The statocyst network was used to reproduce the two types of dynamics observed during routine swimming and during hunting behavior, namely, winner take-all dynamics and winnerless competition. Our simulations show that a model built with conductance-based neurons and realistic inhibitory connections can display both types of dynamics depending on the stimulation from the statolith or from the hunting neuron, respectively. We have also shown that these two dynamics can be used by the wing CPG to generate the characteristic rhythmic motion during routine swimming, and a fast irregular motion that is observed during hunting behavior. The nature of the sensorimotor transformation cannot be described as a simple mapping but as a dynamical process that involves reading one spatio-temporal code (sensory) and translating it into a different spatio-temporal code in the CPG (motor).

*Clione*'s hunting behavior is directed toward locating and capturing prey. The motor strategy during hunting is determined by the difficulty in detecting an odor source in the water. As it has been shown in the behavioral experiments reported in [15], after triggering the hunting, *Clione*'s search movements are not directed by the prey since they continue even when the prey is taken out of the water (see Video S2). In an animal with an undeveloped visual system, such a motor strategy increases the chance of locating and capturing the prey.

Our modeling results suggest that, in spite of the intrinsic irregularity of the switching sensory dynamics (a network phenomena) during hunting, specific activation phase locks in the sensory WLC dynamics are transformed into specific motor events in the wing CPG activity. In this sense, we can consider these sequential activations as coordination patterns inside the irregular statocyst dynamics. This is particularly relevant in the context of a complex intrinsic sensory dynamics that has to be transformed into an effective motor program. Hunting search is highly irregular, but nevertheless organized and coherent. From this perspective, it makes sense that a complex sequential activation of sensory neurons contains coordination cues in the form of activation phase locks that can be interpreted and executed by the motor CPG to generate the motor program. Although we have not addressed it here because of lack of information regarding connectivity, the tail motoneurons could also use cues from the statocyst dynamics to contribute to an effective hunting search in coordination with wing motor activity. Tail movements do not have the repetitive pattern of a CPG output and thus these movements are less restricted and more prompt to be modulated by sensory input.

Beyond the specific role played by the statocysts in *Clione*'s hunting behavior, the results discussed in this work, as well as the experimental results reported in [15], [16] and those obtained by [35] on the pulmonate snail *Lymnaea*, show that the statocysts can perform dual functions depending on the behavioral context. During routine swimming of *Clione*, the statocysts perform a purely sensory function and gravimetric reflexes are used for maintaining a vertical spatial orientation. In contrast, during hunting the statocysts participate in generating a hunting motor program. In both cases, the statocyst output is used to drive a CPG, hence, organizing motor behavior. Under our description, the sensory signals are modified to fit the changing behavioral context. In a sense, the statocyst network is fooled by the hunting neuron. However, this sensory dynamics is interpreted by the rest of the nervous system as during routine swimming, which results in a complex hunting search motor pattern. In spite of its irregularity, the statocyst activity can contain coordination cues to organize a complex motion, i.e., a hunting search program. There are some advantages in generating the motor program right at the sensory network, as the rest of the neurons in the sensory-motor transformation can just react normally to this signaling. Another alternative would be to generate the program at the cerebral ganglia. However a strong experimental fact goes against this hypothesis as fictive hunting search cannot occur without the statocysts [15]. The experimental and modeling results reported in this paper support the view that the dual dynamics of the statocyst network by itself can explain the two motor programs observed during routine swimming and during hunting behavior.

## Methods and Models

### Experimental Methods

Preparations for electrophysiological experiments were made in ice-cold seawater to prevent excitation of nociceptive afferent fibers. The preparation, including cerebral, pedal, and abdominal ganglia with the wing nerves, was pinned to a Sylgard-lined Petri dish as described previously [15]. Extracellular recordings from nerves were made by using glass suction electrodes or stainless-steel electrodes. Intracellular recordings were made using glass electrodes (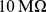) filled with 3 M KCl. The signals were acquired with a Digidata board (Molecular Devices, Union City, CA) and stored for later analysis with Dataview (http://www.st-andrews.ac.uk/wjh/dataview/). The spikes were sorted from the extracellular recordings in Dataview, using threshold and the spike template. Because there was little superposition in spike firing, we could typically sort four or five units in the statocyst nerves.

Fictive hunting behavior was induced by application of physostigmine as in [24] and [15]. To achieve fictive hunting, the seawater covering the isolated nervous system was replaced by seawater containing 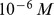 physostigmine.

### Models

All the equations of our models were numerically solved with a Runge-Kutta6(5) variable step method with a maximum error of 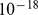.

#### Neuron models

To model the individual behavior of all the cells of our neural networks we have used a well known Hodgkin-Huxley type model [36], proposed by Komendantov and Kononenko (KK) for molluscan neurons [33]. The model consists of eight membrane currents (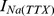, 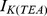, 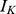, 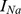, 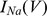, 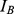, 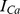 and 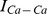) and an intracellular calcium buffer. The details and the equations that describe the dynamics of the KK model can be found in [33].

This is a very rich dynamical model that shows several patterns of activity as a function of the parameters used in the simulations. Here we used the original parameters of the model (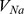 = 40 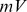, 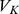 = −70 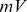, 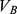 = −58 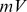, 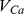 = 150 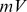, 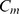 = 0.02 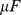, 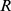 = 0.1 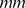, 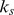 = 50 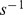, 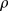 = 0.002, 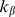 = 15000 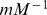 and 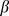 = 0.00004 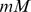), except for the maximal conductances of the channels. All the neurons in a given network (statocyst, cerebral ganglia and wing CPG) have the same parameters. We have adapted these parameters to better match the activity of the *Clione*'s neurons. Following experimental recordings, in isolation, the hunting neuron, the cerebral interneurons and the CPG cells are set in irregular spiking behavior, while the SRCs are set into an irregular spiking-bursting behavior (for details see [18]). The specific parameters used in the simulations reported in this paper are shown in Table 2. However, the results presented in this paper can be easily reproduced with other values.

**Table 2:** Maximal conductances of the ionic channels.

	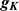	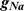	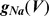	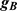	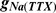	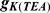	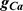	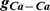
Hunting neuron (H)	0.25	0.0231	0.11	0.1372	400.0	10.0	1.5	0.02
Statocysts cells (SRC1-6)	0.25	0.02	0.11	0.128	400.0	10.0	1.0	0.01
Cerebral ganglia cells (CG1-3)	0.25	0.0231	0.0795	0.1372	400.0	10.0	1.5	0.02
Wing CPG neurons	0.25	0.0231	0.0807	0.1372	400.0	10.0	2.0	0.02

Values of the maximal conductances for each ionic channel in the different neurons of our models. All neurons in the same network have the same parameters. Units are 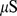.

#### Connection models

In the model there exist three different kinds of connections: inhibitory chemical synapses (represented by filled circles in all the graphical representations of the circuits), excitatory chemical synapses (open circles) and electrical (resistors). Equations used to model each kind of connection are:

- To model inhibitory and excitatory chemical synapses we have used the following equation [37]:
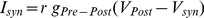
(1)where 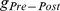 is the maximal conductance of the connection (Table 3 shows the values used in the simulations presented here for each chemical connection); 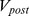 is the postsynaptic potential; 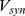 is the synaptic reversal potential (in our simulations 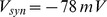 for all the inhibitory and 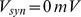 for all the excitatory synapses); and the value of 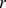 gives the fraction of the open channels in the postsynaptic neuron and it is given by:
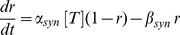
(2)being [T] the concentration of transmitter. To calculate the value of 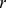 we follow the approach of Destexhe et al. and we assume that 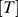 occurs as a pulse (for details see [37]). During these pulses 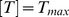. After that 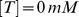. In all our simulations we assume that pulse duration for transmission is equal to 1 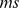 and 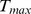 is equal to 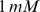 [37]–[39].
  Parameters used to calculate the value of 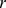 are:
  In the connections between the hunting neuron and the SRCs (see Figure 1): 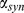 = 2.0 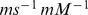 and 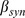 = 0.75 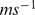

  In connections between two SRC (see Figure 1): 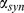 = 1.0 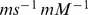 and 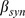 = 0.1 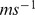

  In connections between a SRC and a cerebral ganglia cell (see Figure 5): 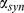 = 1.0 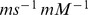 and 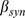 = 0.1 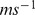

  In the connections between two cerebral ganglia cells (see Figure 5): 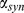 = 2.0 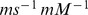 and 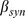 = 0.2 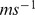

  In connections between a cerebral ganglia and a wing CPG cell (see Figure 5) 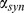 = 1.5 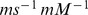 and 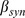 = 0.5 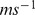

  And, finally, in the connections between the neurons of the wing CPG model (see Figure 5): 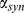 = 2.5 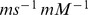 and 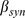 = 1.0 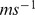 for fast synapses; and 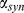 = 2.5 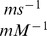 and 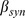 = 0.25 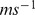 for slow synapses
- Gap junctions:
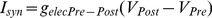
(3)where 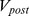 and 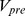 are the post and presynaptic potential.

**Table 3:** Maximal conductances of chemical synapses.

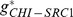	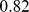	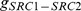	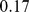	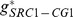	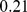	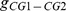	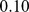	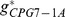	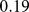
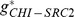	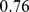	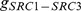	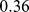	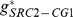	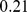	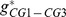	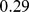	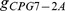	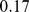
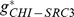	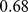	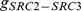	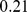	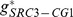	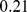	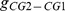	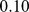	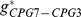	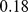
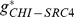	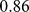	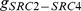	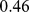	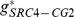	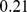	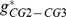	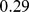	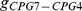	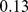
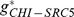	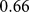	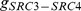	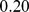	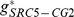	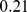	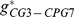	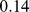	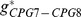	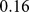
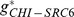	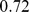	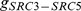	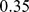	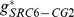	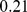	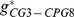	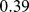	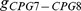	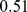
		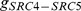	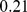					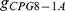	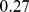
		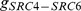	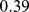					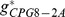	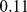
		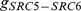	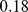					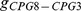	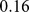
		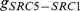	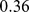					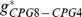	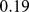
		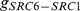	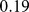					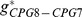	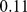
		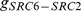	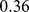					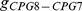	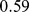

Values of maximal conductances of chemical inhibitory (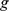) and excitatory (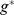) synapses. Units are 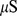.

#### Modeling the statocysts

To model a statocyst we have developed a neural network with six SRCs under the action of a single CHI (Figure 1). As in the biological network [19], in our model all the synapses between SRCs represent inhibitory non-symmetrical connections with different weights. This is an essential feature to achieve winnerless competition dynamics [40], [41]. In a similar manner to the model presented in [28] or [15], all SRCs in the network send and receive two signals to the rest of the network. Connections are established with the next two adjacent cells. Thus, the network can be described as two inhibitory triangles of different synaptic strengths weakly coupled through an inhibitory loop between adjacent neurons. On the other hand, connectivity between the CHI and the SRCs is excitatory [24], [25].

Finally, we model the action of the statolith in a SRC by injecting a constant current (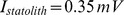) in the receptor that is pressed at a given time. We assume that only one receptor is pressed by the action of the statolith. For the rest of SRCs 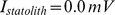.

#### Modeling the cerebral ganglia neurons

Each SRC in the living network is connected through excitatory synapses to a group of cerebro-pedal interneurons in different areas of the cerebral ganglia [25], although the details of these connections are still unknown. Experimental results show a significant correlation between the activity of the SRCs and the wing CPG cells in the biological network (see Figure 5 in [15] and Figure 5 in [16]), suggesting a limited processing role for the cerebral neurons in between. Taking into account this assumption, we have built a simple cerebral interface (CG1, CG2 and CG3) between the sensory network and the motor CPG (see Figure 5). We have divided the SRCs into two groups: the first group (SRC1–3, left side of the statocyst) is connected to the CG1 cell, while the second one (SRC4–6, right side) is connected to the CG2 cell. Note the mutual inhibition between CG1 and CG2. The information received in these neurons is integrated in the CG3 cell. This neuron transduces the dynamics of the statocyst to the wing CPG.

#### Modeling the wing CPG

*Clione*'s movement is driven by a pair of wings and a tail (see pictures in Figure 1 and Videos S1 and S2). Each wing is controlled by a neural network located in the pedal ganglion. This network acts as a CPG generating the beating rhythm of the wing. The morphology and functionality of the CPG network have been described in detail [18], [20]. The wing CPG consists of about 20 cells grouped into two half centers (with about 10 cells in each group): the dorsal and the ventral groups. The first is driven by interneurons of group 7. The latter, by interneurons of group 8. The motoneurons in each group transform the rhythm generated into a motor output. Due to the mutual inhibition in the network, the pacemaker groups 7 and 8 tend to fire in antiphase. This property defines the alternation of the dorsal and ventral phases of a routine swim cycle (Figure 6).

In our models, the wing CPG is a simplified circuit built with six neurons (for details on the living CPG connectivity see [17], [18], [20]): two interneurons (7 and 8) and four motoneurons (1A, 2A, 3 and 4). Each single neuron represents the equivalent electrically coupled groups of cells in the biological circuit. In this way, the rhythm generators are neurons 7 and 8, while neurons 1A, 3, 2A and 4 are the motoneurons that innervate the wing muscles. In all our simulations the cells in a same group fire synchronously. Therefore, we have chosen neurons 1A and 2A as representative cells of the output rhythm.

### Analysis Methods

#### Lyapunov exponents

To characterize the level of irregularity of the activity of our models we calculate the Lyapunov exponents from the model equations [28] describing the system under study. The value of these exponents provides a measure of the irregularity of the system dynamics as they quantify the rate of divergence of nearby trajectories. The existence of a positive Lyapunov exponent means that the system under study is chaotic.

One of the requirements for the model was to generate irregular activity during hunting both in the sensory network and the motor network. This kind of neural activity may be responsible of the generation of *Clione*'s complex search trajectories to locate the prey when hunting behavior is triggered.

#### Phase-locks detection

During hunting behavior, the timing of the SRCs activity is irregular, in fact chaotic as reflected in our model analysis. Nevertheless, there exist time windows of different sizes where the activation sequence among the SRCs is preserved. We call these sequences in the sensory signals “activation phase locks”. To detect the phase locks in the statocyst network activity, we have defined the method that consists of the following steps:

- Transform the voltage time series into discrete temporal sequences 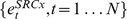 where 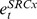 can be 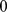 or 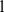. 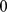 means that at time 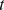 the membrane potential of the SRCx is under a given threshold, in our case 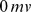. 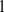 means that is over the threshold (in our case that the membrane potential is positive).
- Map the statocyst activity to activity words 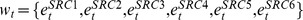. In this manner, we translate the activity of our statocyst model to words of six bits, one bit for the activity of each SRC. For example, the word 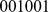 denotes that SRC3 and SRC6 are active at a given time.
- Compress the sequence of activity words by removing identical consecutive words. This compression allows to eliminate the effect of duration differences in the activation periods.
- Search in these compressed series for identical sequences of consecutive words. We only consider sequences with a minimum of four active SRCs (thus, we omit trivial sequences) which occur frequently in the time series.

With this method we transform the phase lock detection into a search for specific words in the discretized time series.

## Supporting Information

Video S1 **This video shows a group of** ***Cliones*** **in the two behavioral states described in the paper: routine swimming and hunting search behavior.** *Cliones* at the top and bottom of the tank display the typical routine swimming. Note the regular beating rhythm of both wings. The *Clione* in the middle displays hunting search behavior and is turning and looping in a faster time scale. The right part of the image corresponds to a mirror reflection that helps to visualize the 3D motion.
(OGG)

Video S2 **This video shows a closed-up of** ***Clione*** **'s hunting behavior.** The hunting trajectory consists of an irregular sequence of loops and turns. Note the difference between the slow and very regular beating rhythm during routine swimming (*Clione* at the bottom of the tank in Video S1) and the fast and non regular beating movements during hunting search behavior.
(OGG)
